# Supplementary material for: Will the California Current lose its nesting Tufted Puffins?
Source: PeerJ. 2018 Mar 22;6:e4519. doi: 10.7717/peerj.4519 (PMC5866916; doi:10.7717/peerj.4519)
Supplement: Supplemental Information 5 [file peerj-06-4519-s005.docx]

**Supplementary Materials**

Hindcast 1950 Results

Coverage gaps in historical survey data on Tufted Puffins makes a robust analysis of hindcast projections difficult. For example, lack of historical data from puffin observations throughout Oregon inhibits crucial input reference points to compare hindcast projections with. For these reasons, in this analysis hindcast projections serve to provide additional insights in to the relationship between Tufted Puffin range and temperature and more site-specific analysis with the available historical data may provide further evidence to the documented effects of temperature on changes in puffin nesting habitat.

Current models projected to the past using historical climate data indicate southern range stability and some expansion, mirroring historical records and estimates of Tufted Puffin range expansion in the southern portion of Tufted Puffin range with past, cooler climate data (Figure S.1) (Hanson & Wiles, 2015). Ensemble projections failed to map suitable habitat in northern and central California but demonstrated expansion, relevant to current-day, in suitable habitat over most of the Washington and Oregon coastline (Figure S.1), again consistent with the influence of temperature on Tufted Puffin range changes in the California Current.

References

Hanson, T. & G. J. Wiles. (2015). Washington state status report for the Tufted Puffin. Washington Department of Fish and Wildlife, Olympia, Washington. 66 pp.

Labay, B., Cohen, A. E., Sissel, B., Hendrickson, D. A., Martin, F. D., & Sarkar, S. (2011). Assessing historical fish community composition using surveys, historical collection data, and species distribution models. *PLoS ONE*, *6*(9), e25145.

Raxworthy, C. J., Martinez-Meyer, E., Horning, N., Nussbaum, R. A., Schneider, G. E., Ortega-Huerta, M. A., & Townsend Peterson, A. (2003). Predicting distributions of known and unknown reptile species in Madagascar. *Nature*, *426*(6968), 837–841.
