# Supplementary figures and images for: Will the California Current lose its nesting Tufted Puffins?
Source: PeerJ. 2018 Mar 22;6:e4519. doi: 10.7717/peerj.4519 (PMC5866916; doi:10.7717/peerj.4519)

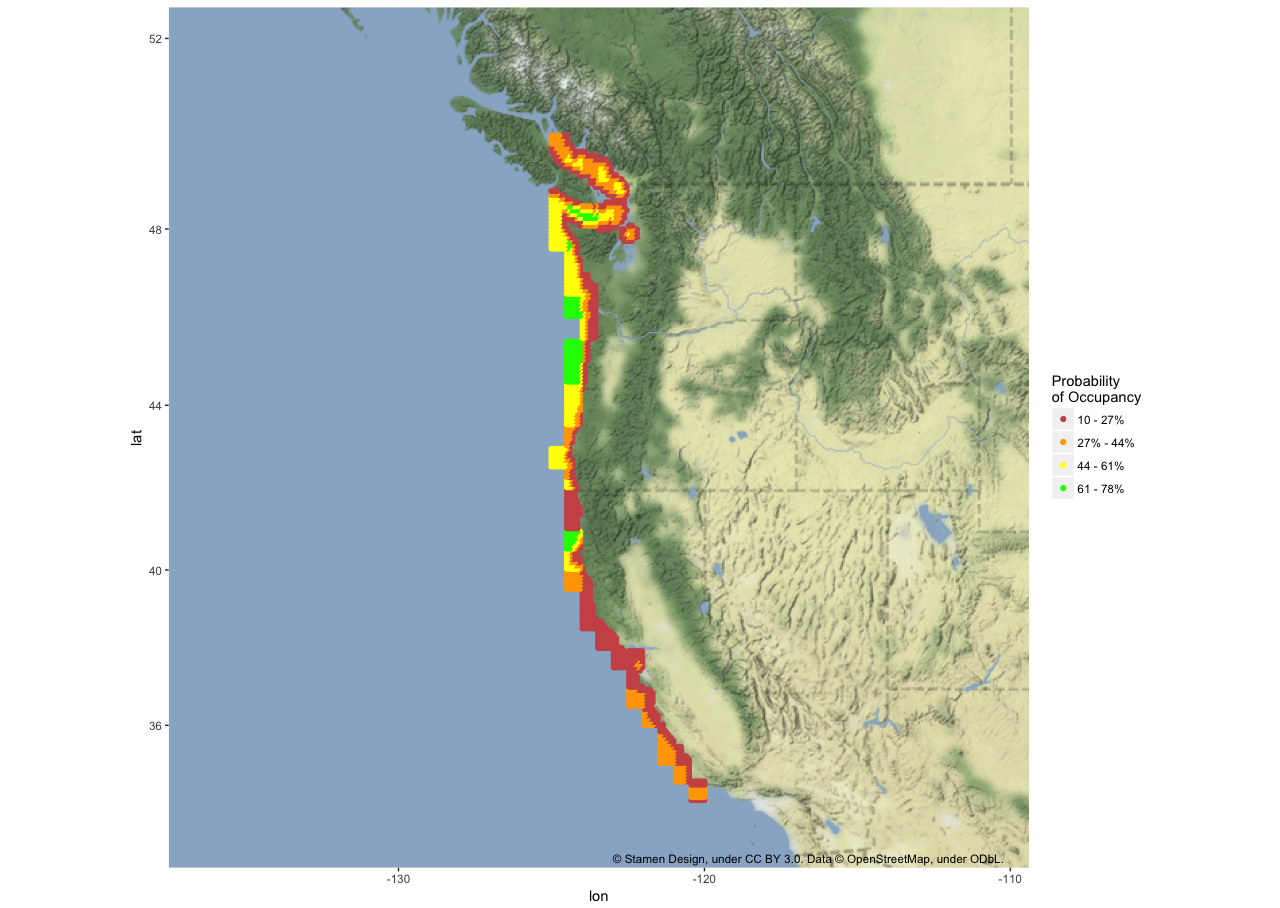

Supplement: Figure S1 — Tufted Puffin breeding habitat range projection map exclusive to California Current (32°N–48.5°N). Probabilistic map, color bins display percent probability of grid cell representing suitable habitat under 1950 climate. Map tiles ©Stamen Design, underCC BY 3.0. Data ©OpenStreetMap, underODbL. [file peerj-06-4519-s006.png]
