# Supplementary material for: Will the California Current lose its nesting Tufted Puffins?
Source: PeerJ. 2018 Mar 22;6:e4519. doi: 10.7717/peerj.4519 (PMC5866916; doi:10.7717/peerj.4519)
Supplement: Table S1 [file peerj-06-4519-s007.docx]

| **Environmental Variable** | **Measurement** | **Unit** |
| --- | --- | --- |
| Annual Temperature Range  (ATR) | Maximum temperature – minimum temperature | °C |
| Mean diurnal range  (MDR) | Mean of monthly (max temp-min temp) | °C |
| Mean temperature of the warmest quarter (MTWQ) | Mean temperature of warmest quarter | °C |
| Annual Precipitation  (AP) | Annual Precipitation | cm |
| Precipitation of the warmest quarter (PWQ) | Precipitation of warmest quarter | cm |
| Distance to ocean  (DIST) | Distance of grid cell to ocean | km |
