# Supplementary material for: Will the California Current lose its nesting Tufted Puffins?
Source: PeerJ. 2018 Mar 22;6:e4519. doi: 10.7717/peerj.4519 (PMC5866916; doi:10.7717/peerj.4519)
Supplement: Table S2 [file peerj-06-4519-s008.docx]

|  | **Climate Data** | **Biological Data** |
| --- | --- | --- |
| **Past Period** | 1910-1950 | Habitat projections |
| **Current Period** | 1950-2000 | 1950-2009 |
| **Future** | 2050 IPCC RCP 4.5, RCP 8.5 | Habitat projections |
